# Acupuncture benefits for Flammer syndrome in individuals with inherited diseases of the retina

**DOI:** 10.1007/s13167-017-0096-4

**Published:** 2017-05-31

**Authors:** Tilo Blechschmidt, Maike Krumsiek, Margarita G. Todorova

**Affiliations:** Department of Ophthalmology, University of Basel, Mittlere Strasse 91, CH-4031 Basel, Switzerland

**Keywords:** Inherited diseases of the retina, Retinitis pigmentosa, Flammer syndrome, Acupuncture, Treatment, Predictive, preventive and personalized medicine

## Abstract

**Background:**

Patients with inherited diseases of the retina (IRD) often exhibit signs and symptoms of Flammer syndrome (FS). Acupuncture treatment has shown its positive effect on visual function in patients with IRD. The aim of the present study is to examine the effect of acupuncture on signs and symptoms of FS in a cohort of patients suffering simultaneously FS and IRD.

**Patients and methods:**

A prospective pilot study was performed on 17 patients with FS and IRD: rod-cone dystrophy, Nr: 12 (RCD); cone-rod dystrophy, Nr: 3 (CRD) and inherited macular dystrophy, Nr: 2 (IMD; 12♀, 5♂; mean age: 44.19 y; SD ±17.09 y). Acupuncture treatment was done applying needle acupuncture of the body and the ears. The treatment was scheduled at 10 half-hour sessions over 5 weeks. Primary outcome was evaluation of the post-acupuncture effect on the signs and symptoms of FS in IRD patients using multiple-choice questionnaires.

**Results:**

Following acupuncture, we found improvement in signs and symptoms of FS in patients suffering simultaneously IRD, as for instance (Nr. patients: improvement/suffering/total): a reduced tiredness (10/11/17), shorter sleep onset time (10/11/17), warmer feet and hands (10/10/17) and reduced frequency of headache attacks (9/11/17). Surprisingly, in four RCD patients and in one IMD patient, a reduction of macular edema was documented.

**Conclusions:**

The applied acupuncture protocol for FS in IRD patients showed improvement in FS signs and symptoms and was tolerated well. Nevertheless, the objective evaluation of this complementary therapy on FS in IRD patients remains to be elucidated.

## Background

Inherited retinal dystrophies (IRDs) are a genetically and phenotypically heterogeneous group of diseases, characterized by progressive reduction of rod and/or cone photoreceptor cell function [1–3]. The genetic heterogeneity in IRDs reflects the variable vulnerability of photoreceptor cells to a variety of environmental, intracellular and extracellular factors [4].

Flammer syndrome (FS) refers to a predisposition to react differently to a variety of stimuli, such as coldness and physical or emotional stress [5, 6]. The most prominent sign in FS is the dysregulation of blood vessels, which has also been discussed as an accompanying feature in a subgroup of IRD patients, namely in the rod-cone dystrophy (RCD) patients [7–9]. Of particular note in RCD patients is the fact that they often suffer symptoms and signs of FS [5, 8, 10, 11]. In fact, the cohort study of Konieczka et al. confirmed 7 out of 15 signs and symptoms of FS to occur more often in RCD patients than in the normal population [9]. Furthermore, in RCD patients, the visual impairment is accompanied with additional factors, such as disturbed ocular blood flow, discussed to influence the progression of the degeneration [12–16]. Taking all of the discussed, the presence of FS in patients with RCD seems to be a predictor for disturbed blood flow regulation.

IRDs are among the leading causes of visual impairment or blindness early in life and are the leading cause of legal blindness among children and working-age adults [17, 18]. Therefore, in order to improve vision of IRD patients, therapeutic approaches are under investigation, among which pharmacotherapy, neuroprotection, gene therapy, stem cell therapy, optogenetics and retinal prostheses are included [19].

The acupuncture treatment is still considered a non-mainstream therapeutic approach. Yet, its positive effect has been shown in a variety of neuro-degenerative and psychosomatic diseases, following cerebral and peripheral ischemia. Acupuncture treatment has also been effectively used to improve blood flow. Studies on electro-acupuncture in rabbits with vertebro-basilar insufficiency showed improvement in their vestibulo-ocular reflex, through improvement of the basilar artery hemodynamic, inner ear blood flow and blood viscosity [20]. Moreover, traditional acupuncture stimulation has shown its positive effect on systemic blood flow, an effect, which supposedly is, in part, mediated by the central nervous system [21]. Acupuncture has also been applied in various diseases involving psychosomatic status, such as anxiety, depression, sleep disturbances [22]. In a depression rat model induced by chronic stress, acupuncture has shown its positive effect on regulation of circadian rhythm, temperature and nocturnal melatonin secretion [23]. Furthermore, in a mice model of Parkinson’s disease, the application of electro-acupuncture has proven to be effective in slowing the degeneration of dopaminergic neurons in the ventral midbrain [24].

Previous reports on the application of acupuncture in patients with RCD have shown improvement in visual function, as measured by static/ kinetic perimetry, dark adaptation, electrooculogram, contrast sensitivity [25, 26] a finding we confirmed in our RCD patients, but also in a variety of IRD patients treated with needle acupuncture of the body and the ears [27]. Surprisingly, in the majority of our IRD patients, we mentioned signs and symptoms related to FS. Thus, we assume, the presence of FS to be a predictive finding for disturbed blood flow.

As the quality of life of patients with IRD is greatly dependent on fluctuations in the remaining central and peripheral vision, and also on disturbed blood flow, any attempt to prevent and to stabilize ocular and systemic blood flow seems to be of benefit for the patient. Following that, and as IRD patients suffer often simultaneously from FS [5, 8, 9, 27], the application of acupuncture might be a promising alternative to the conventional treatment.

With this background in mind, we aimed to examine the effect of needle acupuncture on the signs and symptoms of FS in patients suffering simultaneously IRD and FS using a multiple-choice questionnaire. In addition, the specific sign or symptom of FS for which the acupuncture treatment was more beneficial for the IRD patient was evaluated.

## Materials and methods

An observational pilot study, in which all subjects received acupuncture, was performed. We aimed to determine the efficacy or proof of principle with the standardized acupuncture protocol. All patients were recruited through the Department of Ophthalmology of the University of Basel (TMG; BT).

The acupuncture protocol was developed for a group of retinal diseases based on the extensive clinical experience of an ophthalmologist and at once licensed and qualified acupuncturist (BT). A placebo control group was not included in this pilot study, as it remains controversial.

All procedures took place at the acupuncture unit at the Department of Ophthalmology of the University of Basel between November 2014 and January 2017. The study and data accumulation were in conformity with institutional requirements, and in accordance with the statements and principles of the Declaration of Helsinki, as well as all governmental regulations. All subject signed informed consent before participation in the study.

### Subjects

Seventeen patients suffering simultaneously from FS and IRDs followed up at the diagnostic unit of the Department of Ophthalmology (University of Basel, Switzerland) were enrolled for the study. According to the clinical phenotype and electrophysiological and genetic findings, patients were divided into the following groups:

- Patients with phenotypic characteristics of rod/rod-cone dystrophy (RCD), *N* = 12 (24 eyes);
- Patients with phenotypic characteristics of cone/cone-rod dystrophy (CRD), *N* = 3 (6 eyes);
- Patients with phenotypic characteristics of inherited maculopathy (IMD), *N* = 2 (4 eyes).

Inclusion criteria for all patients were: phenotypical and electrophysiological picture of IRD; positive history of a FS, as well as a Caucasian origin. Ten multiple-choice items for signs and symptoms of FS, reported with increased frequency in RCD patients [9], were included in our a questionnaire: cold extremities, low blood pressure, low body mass index, prolonged sleep onset time, reduced feeling of thirst and increased sensitivity in general, e.g. increased sensitivity to certain drugs, increased pain sensitivity and increased sense of smell (Table 1). The IRD patient was defined as suffering simultaneously FS if he/she answered 7 or more out of 10 questions with “yes”.

**Table 1:** Signs and symptoms of Flammer syndrome in patients with IRD. The multiple-choice questionnaire consisted of 10 items with the following choices: “often”, “never” or “I do not know”. The mid-sided column shows how often our IRD patients exhibited sign or symptom of FS. The right hand-side column represents how many IRD patients suffering simultaneously signs or symptoms of FS improved following acupuncture treatment

**Evaluated signs and symptoms of Flammer syndrome in our IRD patients**	**Present before acupuncture (Nr. patients: suffering/total)**	**Reduced after acupuncture (Nr. patients: showing improvement/suffering)**
Cold hands and/or feet	10/17	10/10
Low blood pressure	11/17	10/11
Low body weight	10/17	5/10
Prolonged sleep onset time	11/17	10/11
Reduced feeling of thirst	10/17	9/10
Increased sensitivity (smell and pain sensation, response to certain drugs)	9/17	7/9
Migraines and headaches	11/17	9/11
Tinnitus	10/17	8/10
Perfectionism	11/17	2/11
Reversible skin blotches (red or white)	10/17	9/10

Exclusion criteria were: above inclusion criteria not fulfilled; absence of signs and symptoms of FS; presence of ocular and/or systemic pathology other than IRD; currently under antidepressants, alcohol or drugs; unwillingness to participate in the study, general or local hyper-reactivity to acupuncture treatment.

All IRD patients, fulfilling our inclusion criteria, before starting and following acupuncture treatment, underwent detailed ophthalmic examination including: refraction, best corrected visual acuity (BCVA, standard decimal visual acuity ETDRS charts), contrast vision (CSV-1000, Vector Vision), intraocular pressure (Goldmann tonometer), slit-lamp examination, biomicroscopy and fundoscopy. In addition, IRD patients with stable fixation performed visual field examination also on Goldmann perimeter (Haag-Streit; for detailed data, please refer to our previous publication [27]).

**Fig. 1:**
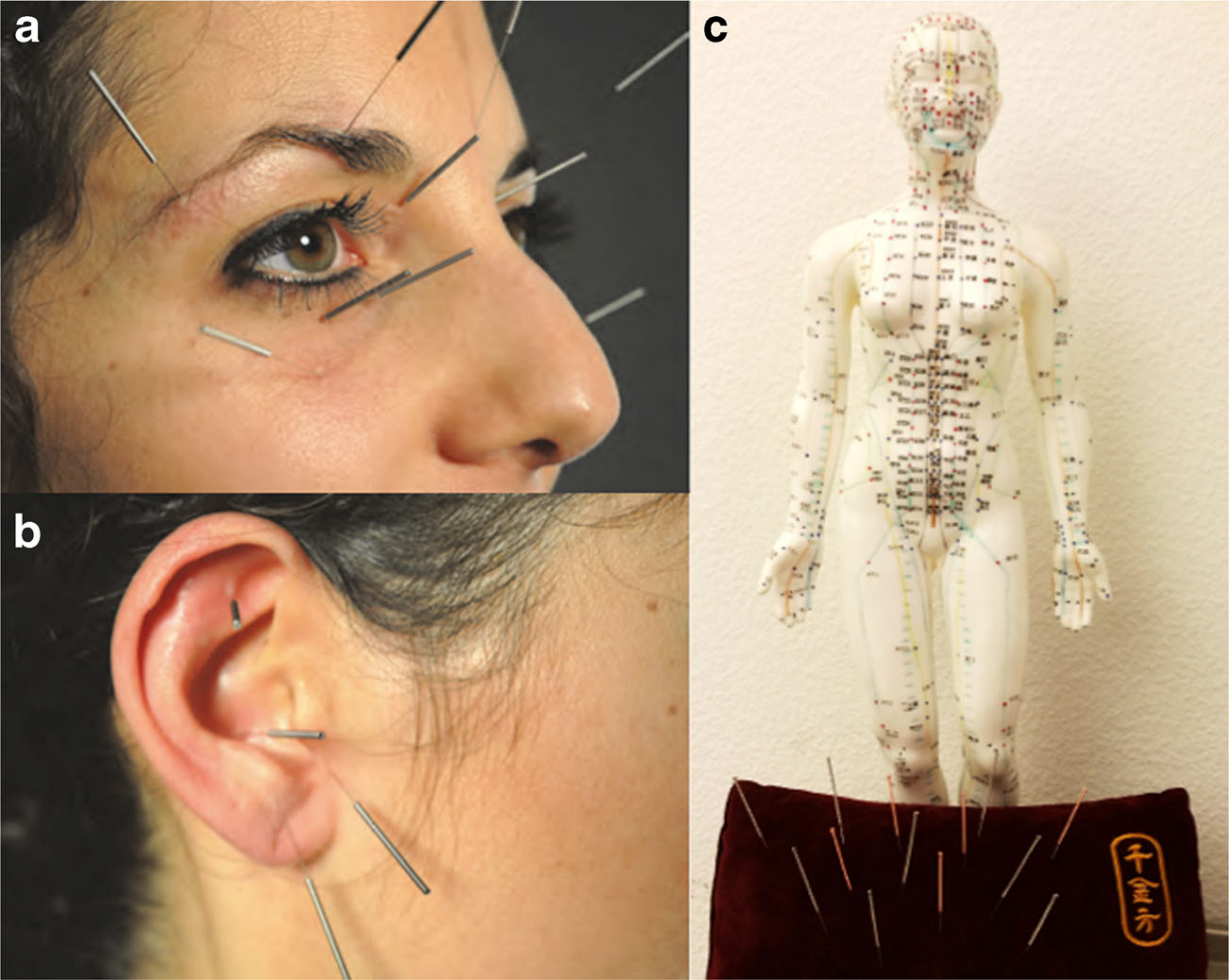
Representative picture of acupuncture treatment of the peri-orbital region (**a**), of the ears (**b**) and the body (**c**). For our IRD patients suffering simultaneously FS, the treatment was scheduled at 10 half-hour sessions over 5 weeks. All acupuncture points, the alternation in treatment, as well as the stimulation duration are listed in Table 2

The acupuncture method followed a standardized protocol, as described in our previous study [27]: The acupuncture protocol consists of 10 sessions of 30-min duration administered twice a week over a period of 5 weeks (Fig. 1; Table 2).

**Table 2:** Acupuncture points. Standardized needle acupuncture protocol was applied as shown in Fig. 1. All acupuncture points, the alternation in treatment (in Italic), as well as the stimulation duration are listed in the table

**Acupuncture study protocol**			Laterality
Treatment modality	Treatment 1 (a)	Treatment 2 *(b)*	
Needle Nr:	Alternating with treatment 2	Alternating with treatment 1	
1	GV20 (Bai Hui)		UL
2	CV6 (Qi Hai)		UL
3	UB23 (Shen Shu)		BL
4 (a/*b*)	UB18 (Gan Shu)	*UB20 (Pi Shu)*	BL
5	GB20 (Feng Chi)		BL
6	LI4 (He Gu)		BL
7 (a/*b*)	TE5 (Wai Guan)	*SI3 (Hou Xi)*	BL
8	LV3 (Tai Chong)		BL
9 (a/*b*)	GB37 (Guang Ming)	*KI3 (Tai Xi)*	BL
10 (a/*b*)	SP6 (San Yin Jiao)	*ST36 (Zu San Li)*	BL
11	UB1 (Jing Ming)		BL
12 (a/*b*)	ST1 (Cheng Qi)	*EX-HN7 (Qiu Hou)*	BL
**Additional ear acupuncture points (alternately, starting with the right ear)**			
13	Eye point (24a)		UL
14	Liver zone (97)		UL
15	Kidney zone (95)		UL
16	Heart zone (100)		UL
17	Thalamus point (26a)		UL
**+ One semi-permanent needle (Press Tack Needle; alternately, starting with the left ear, points in the order specified):**			
14	Liver zone (97)		UL
15	Kidney zone (95)		UL
17	Thalamus point (26a)		UL
**Needle stimulation (after 15 min) at following points:**			
6	LI4 (He Gu)		BL
8	LV3 (Tai Chong)		BL
2	CV6 (Qi Hai)		UL
13–17	Ear points		UL
**Duration of needle stimulation:**			
30 min			

Each patient was asked to complete a questionnaire concerning his/her general condition/accompanying disease and ophthalmic disease before the first and after the last acupuncture treatment. In addition, before and following acupuncture treatment, all patients completed a questionnaire assessing subjectively for presentation of sign and symptoms of FS (Table 1).

The scheduling of each patient and the complete orthoptic examinations were performed by the same experienced orthoptistin (KM). Before the initial appointment for treatment, the acupuncturist gave the patient a brief introduction outlining the duration and the course of treatment, as well as the possible complications (BT). Each scheduled treatment session was initiated only after a short welcome of the patient and questions concerning his/her general and ophthalmic condition after the previous treatment, as well as his/her actual general condition.

### Needle acupuncture of the peri-orbital area, of the ears and the body

Needle acupuncture of the peri-orbital area, of the ears and the body was applied as follows: Sterile and disposable single-use needles of different sizes were used, namely Seirin B type needle no. 3 (0.20) x 15 mm, no. 5 (0.25) x 40 mm, no. 8 (0.30) x 30 mm, Seirin Pyonex Press needles P type 0.22 x 1.6 (Seirin Corporation, Shizuoka, Japan); Dong Bang needle DB106 (0.20) x 15, DB105G (0.20) x 25, Dong Bang Press needles 0.20 x 2 x 1.0 (Dong Bang Acupuncture, Inc., Chungnam, Korea). The established protocol indicates the specific preselected points for all participants, needling depths and manipulation techniques. The needles were applied by the same acupuncturist (BT). The standard points for all subjects are located around the eyes, on the head, ears, back, abdomen, arms, hands, lower legs and toes and include: GV-20 (Bai Hui), CV-6 (Qi Hai), UB-18 (Gan Shu), UB-20 (Pi Shu), UB-23 (Shen Shu), GB-20 (Feng Chi), LI-4 (He Gu), TE-5 (Wai Guan), SI-3 (Hou Xi), LV-3 (Tai Chong), GB-37 (Guang Ming), KI-3 (Tai Xi), SP-6 (San Yin Jiao), ST-36 (Zu San Li); local points: UB-1 (Jing Ming), ST-1 (Cheng Qi), ExHN-7 (Qiu Hou); ear points: eye point (24a), liver zone (97), kidney zone (95), heart zone (100) and thalamus point (26a). The needles were applied according to a standardized protocol (Fig. 1, Table 2). Individual choice of acupuncture points was not allowed in contrary to common Chinese medicine (CM). Likewise, due to standardization, the amount of applied needles exceeded the common practice of CM. The locating of needles was performed due to the standards of CM. It was aimed to produce the irradiating needle sensation (`de qi`), if possible. The needles at LI-4 (He Gu), CV-6 (Qi Hai), LV-3 (Tai Chong) and all ear needles were manually stimulated once in each session after 15 min (+/−5 min). Additional influencing techniques, like electro stimulation, heat lamps, music during treatment etc., were not applied.

### Statistical analysis

Acupuncture effect on signs and symptoms of FS was analyzed at the beginning and at the end of acupuncture. The 10 multiple-choice items in the questionnaire could be answered qualitatively as: “often”, “never”, or “I do not know”. In addition, before acupuncture procedure we analyzed for signs and symptoms of FS. We evaluated thereafter to what extend they disappear following the treatment.

## Results

All IRD patients included in the study exhibited simultaneously a clinical picture of FS: RCD, 12 patients (24 eyes); CRD, 3 patients (6 eyes); IMD, 2 patients (4 eyes). Our RCD patients’ (9♀ and 3♂) age ranged from 23 to 71 years (mean: 40.14), our RCD patients’ (2♀ and 1♂) age ranged from 19 to 25 years (mean: 44.42), and our IMD patients’ age ranged from 62 to 72 (1♀ and 1♂).

Objective and subjective improvement in visual acuity, contrast vision and Goldmann visual field following acupuncture was seen in all IRD patients (for details, please refer to our previous publication [27]).

All our IRD patients had pre-acupuncture treatment signs and symptoms of FS, such as cold extremities (10/17 patients; 58.82%), low blood pressure (11/17 patients; 64.70%), low body mass index (10/17 patients; 58.82%), prolonged sleep onset time (11/17 patients; 64.70%), reduced feeling of thirst (10/17 patients; 58.82%); migraine/headache attacks (11/17 patients; 64.70%) and increased sensitivity in general (9/17 patients; 52.94%), e.g., increased sensitivity to certain drugs (9/17 patients; 52.94%), increased pain sensitivity (9/17 patients; 52.94%) and increased sense of smell (9/17 patients; 52.94%; Table 1).

Following acupuncture treatment, general symptoms related to FS improved qualitatively in all our IRD patients. For instance, prolonged sleep onset time (10 patients; 90.90%), feeling cold (10 patients; 100%) and migraine headache attacks (9 patients; 81.81%) were reduced significantly. However, other symptoms such as perfectionism and low body weight, even with significant prevalence pre-acupuncture, did not improve post-treatment (Table 1).

As an example, we provide the data of ophthalmological and systemic subjective and objective findings of a few IRD patients with FS who participated in the study.

A 62-year-old artist lady was referred by her husband for ophthalmological evaluation, due to newly reported reduced visual acuity, color and contrast vision of her right eye. Ophthalmological examination and diagnostic imaging confirmed the diagnosis of Best vitelliform macular dystrophy. In addition, the patient explained suffering sleep disturbances, with prolonged sleep onset time and approximate total sleep duration of 2–3 h. Following applied acupuncture treatment, her BCVA improved from 0.8/0.32 (RE/LE) to 1.0/0.5 (RE/LE). She gained also in contrast vision from 22/11 to 30/21. Evaluated by the OCT imaging, her central vitelliform lesion on the right eye resolved. In addition, her subjective symptoms of cold extremities, increased general and smell sensitivity and increased sleep onset time all reduced notably. She also reported on significant improvement of her sleep quality, as for instance: falling asleep faster, increased total sleep time and significantly improved quality of sleep.

**Fig. 2:**
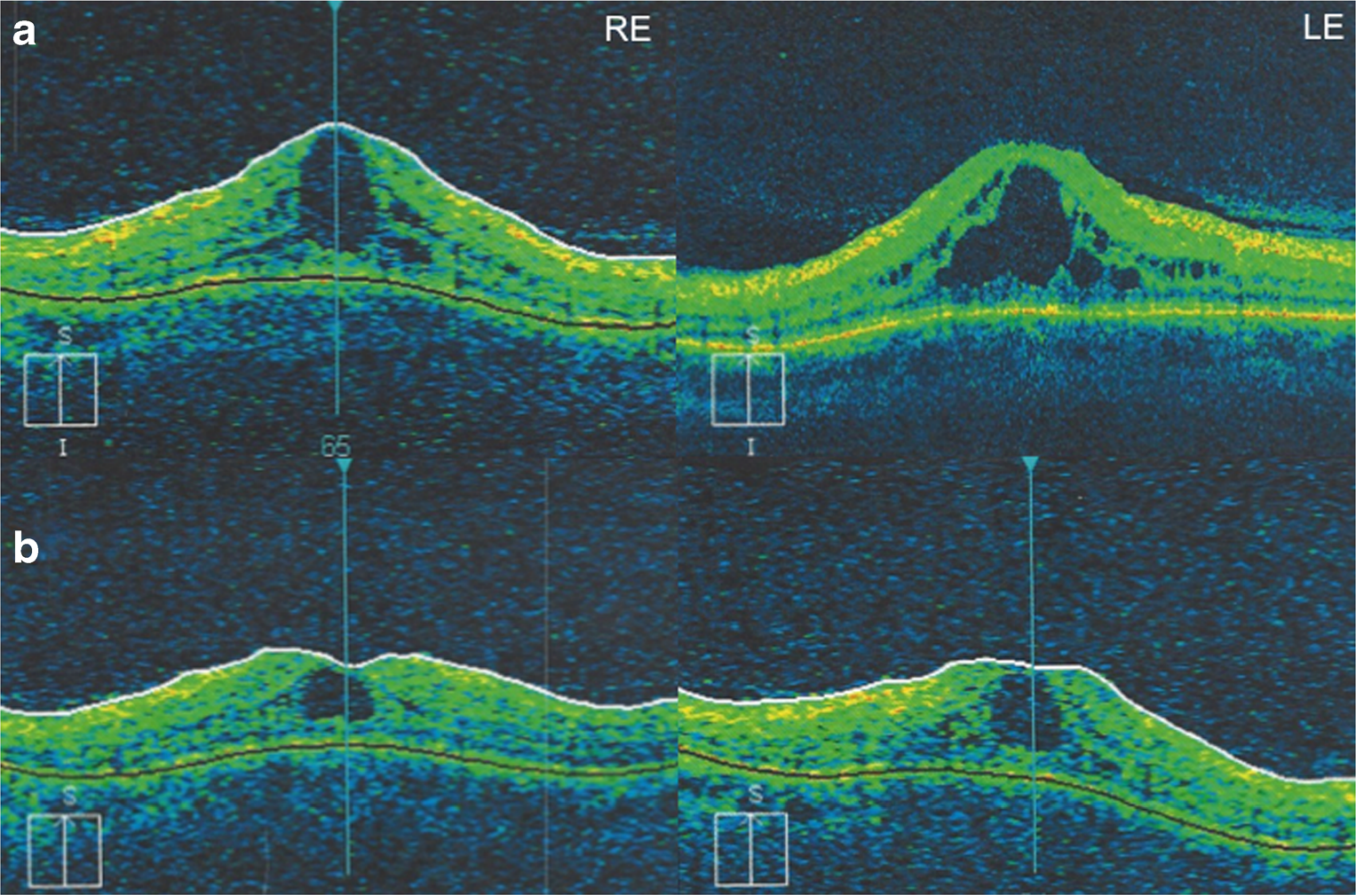
Optical coherence tomography (OCT) imaging (Carl Zeiss Meditec. Dublin, CA, USA) of an RCD patient with irreversible macular edema (**a**). Macular thickness protocol (macular cube 512 x 128) confirms significant reduction of the macular edema following acupuncture treatment (**b**)

A 30-year-old photographer was followed-up because of years in the clinic due to retinitis pigmentosa. Following acupuncture treatment, he reported on improvement of his peripheral vision, but also of his contrast vision. The latter was mentioned in regards to not only daily life, but also while taking photographs in the mountains. His clinical examinations revealed not only improvement in his BCVA from 0.4/0.4 to 0.5/0.5 (RE/LE), in contrast vision from 6/7 to 10/14 (RE/LE), but also reduction of macrocystic macular edema (both eyes), as well documented in Fig. 2.

A 20-year-old gentleman was referred for clinical evaluation to rule out any contrast sensitivity and color vision disturbances, as he failed on his exam for being a painter. Ophthalmological evaluations revealed pre-acupuncture reduced BCVA of 0.8/ 0.5 (RE/LE), contrast sensitivity reduction of 22/10 and blue-axis color vision disturbances, as well as a picture of RP with clinical appearance of macular edema. Several treatment modalities including oral application of carbo-anhydrase inhibitors failed to reduce his macular edema. Following acupuncture treatment, the persistent macular edema showed significant reduction, leading to improvement of his BCVA to 1.0/1.0 and of his contrast sensitivity to 26/14, as well as reduction of his blue-axis color vision discrimination.

A 32-year-old lady with genetically confirmed Usher syndrome type 2 reported on gain in visual acuity and on improvement in visual field following acupuncture treatment. In addition, her sleep disturbances, migraine headache attacks and feeling of cold extremities were notably reduced.

No ocular or systemic adverse effects were reported from any of our IRD patients. Furthermore, all patients included in the study indicated a willingness to repeat the acupuncture treatment.

## Discussion

Results of our pilot study confirmed again that RCD patients [7–9], as well as patients with other IRDs, suffer simultaneously signs and symptoms of FS [27]. A novel finding in the present study is the fact that following standardized acupuncture protocol, all our IRD patients suffering simultaneously FS showed improvement of signs and symptoms related to FS.

### FS and IRDs

Ocular blood flow dysregulation as part of FS has already been discussed in the pathogenesis of ophthalmic-related pathologies [10, 28–30]. Interestingly, our IRD patients featured signs and symptoms related to FS. Therefore, we assume that patients with FS and IRD also suffer insufficient and disturbed blood flow autoregulation. We hypothesize that this could be as a consequence of the following:

With progression of the outer retinal degeneration, apoptosis of the outer retina occurs with secondary remodeling of the inner retina, as well as of the retinal and choroidal vasculature, manifested clinically with disturbed retinal and choroidal blood flow [31]. In such altered conditions, as a consequence of reduced demand for supply, the blood-retinal barrier is compromised and the blood flow is reduced.

ET-1 has already been discussed to be involved in the regulation of retinal vessel size, but also to influence the blood-retinal barrier [6]. In the eye, ET-1 is though to have a greater impact in ocular blood flow regulation [32–35]. In general, ET-1 is upregulated as a result of tissue hypoxia and/or inflammation. Its pro-apoptotic role is supported further by the observation on blockage of axoplasmic transport, on reduction of blood-retinal barrier and ocular blood flow [10, 11]. However, ET-1 has a major impact on the vascular smooth muscle cells only in case of direct access, as for instance, when the blood-retinal barrier is compromised. In fact, in patients with IRDs, the retinal blood flow is insignificantly affected, as long as the blood-retinal barrier is intact [12–16]. Of particular interest is the fact that a significant increase in ET-1 plasma levels in RCD patients, compared to controls, has been found in a number of studies [8, 13, 32, 33, 36]. Interestingly, subjects with FS showed also increased ET-1 plasma levels [5, 6, 8, 45]. Furthermore, in a group of RCD patients, we found a strong association between increased ET-1 plasma levels and positive history for signs and symptoms consistent with FS, supposing thus the contribution of FS on ET-1 plasma levels increase in RCD patients [8]. Therefore, we concentrated in the present study on evaluating the effect of needle acupuncture on the signs and symptoms of FS only on those IRD patients which exhibited signs and symptoms of FS.

FS [5, 6], previously called primary vascular dysregulation (PVD) [10, 11], refers to a predisposition to react differently to a number of stimuli, such as coldness, physical or emotional stress and systemic medication [11, 29, 37, 38]. The most prominent sign is the inborn disturbed blood flow autoregulation [10, 11, 28]. FS subjects often suffer from systemic hypotension [11, 39, 40] showing a completely different pattern of blood pressure variations in association with changes in distal skin blood flow than unaffected persons [40]. The lower blood pressure is also the reason why FS subjects require longer time to fall asleep, especially when they are cold, as warm feet are generally a prerequisite for falling asleep [38, 40, 41]. Here, the increased plasma ET-1 level is discussed as a major factor in the pathogenesis of the described signs and symptoms associated with FS as: reduced thirst sensation [42], atypical headaches and migraines [43, 44] and increased pain sensation [45]. FS occurs more often in females than males, in subjects who are slim more than those who are obese [46], in academics more than in blue-collar workers, and in Asians more than in Caucasians [5, 6, 10]. A concept of FS in IRD patients in the presence of altered blood flow has been proposed in previous works [7, 9, 27].

### Improvement in FS signs and symptoms following acupuncture treatment in patients with IRD

At present, we are still in the early stage of research in this field. Interestingly, all IRD patients included in the present study could benefit from acupuncture treatment by reducing the signs and symptoms related to FS. We suggest that this may be explained as follows:

Therapeutic and preventive modalities to support visual function and to reduce ocular blood flow disturbance in IRD patients suffering FS are crucial. The traditional acupuncture stimulation has shown positive effect on systemic blood flow, an effect which is supposed to be mediated in part by the central nervous system [21]. As IRD patients suffer altered systemic and ocular blood flow [7, 12], it may, in turn, contribute to the presence of symptoms and signs of FS [7, 9, 27]. Thus, we hypothesized that traditional acupuncture of the ears and the body would show improvement of FS signs and symptoms. In agreement, all our IRD patients with FS could benefit from acupuncture, where a variety of signs and symptoms related to FS were notably reduced. As IRD patient simultaneously suffer from systemic blood flow disturbances [5, 8], the positive stimulation effect of acupuncture on systemic blood flow might be expected, and could, in part, explain our results. Our hypothesis is supported by the acupuncture benefit on systemic blood flow, already shown in previous study and discussed as mediated through the central nervous system [21].

We do not know, but, indeed, reduced systemic oxidative stress, due to improved systemic blood flow and, thus, improved oxygen supply, seem to be additional plausible factors for improvement of general symptoms related to FS. Supportive of this is the fact that not only symptoms and signs of FS were reduced notably, but also those observed in several IRD patients with macular edema that had persisted since years were resolved or reduced simultaneously following acupuncture. The latter could be a explained as a consequence of improved ocular-blood perfusion, stabilized ocular-blood barrier and reduced intraretinal oxygen tension, and, thus, reduced retinal oxidative stress in patients with IRD [47]. On the other hand, as the retinal vessels are not innervated, they are not autoregulated by the central nervous system. Here, the local effect of acupuncture on retinal and choroidal vessels in IRD patients with FS could again be related to the reduction of the oxidative stress due to stabilization of the blood flow and, consequently, of the oxygen supply. Therefore, IRD patient with FS may probably benefit from acupuncture treatment by stabilizing both the disturbed systemic and ocular blood flow. However, reduced macular edema following acupuncture may, in turn, be explained as a consequence of reduced retinal venous pressure, as increased venous pressure has been discussed in the pathogenetic chain of events of FS [48–50]. More precisely, in patients with simultaneous presentation of IRD and FS, increased venous pressure may probably lead to an increase in the transmural pressure and, thus, to increased risk of macula edema. Therefore, any possible reduction of retinal venous pressure following acupuncture seems to be of benefit for FS in the IRD patient.

It is worthy of note, however, that 2 out of 10 symptoms and signs related to FS, as perfectionism and low body weight, even if with significant prevalence in our IRD, did not improve much following acupuncture. This is not an unexpected finding, based on the limited number of patients and on the subjective/qualitative statistical approach of the data evaluation. Also, the type of personality is not expected to change much following a complementary therapeutic approach.

## Conclusions

Based on the results of the present study, signs and symptoms of FS could also be mitigated by needle acupuncture of the body and the ears. The applied acupuncture protocol in IRD patients simultaneously suffering FS was tolerated well and showed improvement in FS signs and symptoms.

Nevertheless, treatment modalities known to be crucial in improvement of signs and symptoms of FS, as: ginco biloba, omega-3 fatty acids, low-dose calcium channel blockers and magnesium treatment [51–54], remain of primary choice. Here, acupuncture could serve as an adjunctive approach to potentiate the effect of other therapies.

At present, we still do not know which IRDs patients with simultaneous presentation of FS might benefit more from acupuncture treatment. Nevertheless, the objective evaluation of this complementary therapy on FS in IRD patients remains to be elucidated. Here, evaluation of a larger cohort of patients with simultaneous presentation of FS and IRDs is mandatory. This should include different acupuncture modalities, a quantitative statistical approach and genotyping of patients in order to objectively elucidate the effect of acupuncture on several aspects of FS in the heterogeneous group of IRDs.

## Abbreviations

FS: Flammer syndrome
IRD: Inherited retinal disease
RCD: Rod-cone dystrophy
CRD: Cone-rod dystrophy
IMD: Inherited macular dystrophy
